# Histological and Immunohistochemical Revision of Hepatocellular Adenomas: A Learning Experience

**DOI:** 10.1155/2013/398308

**Published:** 2013-02-28

**Authors:** S. Fonseca, D. Hoton, S. Dardenne, L. Annet, C. Hubert, S. Godecharles, A. Jouret-Mourin, R. Reding, J. B. Otte, J. Rahier, J. F. Gigot, C. Sempoux

**Affiliations:** ^1^Service d'Anatomie Pathologique, Cliniques Universitaires Saint-Luc, UCL, Avenue Hippocrate, 10, 1200 Brussels, Belgium; ^2^Service de Chirurgie, Cliniques Universitaires Saint-Luc, UCL, 1200 Brussels, Belgium; ^3^Service de Radiologie, Cliniques Universitaires Saint-Luc, UCL, 1200 Brussels, Belgium

## Abstract

Light has been shed on the genotype/phenotype correlation in hepatocellular adenoma (HCA) recognizing *HNF1**α***-inactivated HCA (H-HCA), inflammatory HCA (IHCA), and **β**-catenin-activated HCA (b-HCA). We reviewed retrospectively our surgical HCA series to learn how to recognize the different subtypes histopathologically and how to interpret adequately their immunohistochemical staining. From January 1992 to January 2012, 37 patients underwent surgical resection for HCA in our institution. Nine had H-HCA (25%) characterized by steatosis and loss of L-FABP expression; 20 had IHCA (55.5%) showing CRP and/or SAA expression, sinusoidal dilatation, and variable inflammation; and 1 patient had both H-HCA and IHCA. In 5 patients (14%), b-HCA with GS and **β**-catenin nuclear positivity was diagnosed, two already with hepatocellular carcinoma. Two cases (5.5%) remained unclassified. One of the b-HCA showed also the H-HCA histological and immunohistochemical characteristics suggesting a subgroup of **β**-catenin-activated/*HNF1**α***-inactivated HCA, another b-HCA exhibited the IHCA histological and immunohistochemical characteristics suggesting a subgroup of **β**-catenin-activated/inflammatory HCA. Interestingly, three patients had underlying vascular abnormalities. Using the recently published criteria enabled us to classify histopathologically our retrospective HCA surgical series with accurate recognition of b-HCA for which we confirm the higher risk of malignant transformation. We also underlined the association between HCA and vascular abnormalities.

## 1. Introduction

Hepatocellular adenomas (HCA) are rare benign tumors most frequently observed in women on oral contraception [1, 2]. HCA can occur in men on anabolic steroids [3] or be associated with underlying metabolic diseases such as glycogen storage disease [4]. Some associations have also been described with congenital vascular abnormalities of the liver [5–8].

The existence of four different categories of HCA was recently recognized, and the clinical relevance of subtyping these liver lesions according to histological and immunohistochemical features and to molecular alterations was demonstrated [9–15].

*HNF1*α**-inactivated HCA (H-HCA) are associated with *HNF1*α** inhibiting mutations leading to the loss of expression of liver fatty acid binding protein (L-FABP) within the lesion as compared with the surrounding liver parenchyma by immunohistochemistry (IHC). These HCA are histologically associated with marked liver steatosis and do not show cytological abnormalities. The second group, the more frequent, is the inflammatory type of HCA (IHCA) associated with the activation of inflammatory pathways, and showing expression of serum amyloid A (SAA) and C-reactive protein (CRP) by IHC. Histologically, IHCA exhibit variable amounts of sinusoidal dilation, inflammation, and ductular reaction. The third subgroup is related to activating mutation of the *β*-catenin pathway (b-HCA) and carries a higher risk of transformation in hepatocellular carcinoma (HCC). The b-HCA can be identified on IHC by a nuclear accumulation of *β*-catenin and by the presence of a strong cytoplasmic staining for glutamine synthetase (GS). The last group corresponds to the unclassified and less-well understood HCA.

Our aim was to review our series of HCA in light of the new histological and immunohistochemical criteria [11–13, 15] in order to learn how to deal with these lesions prospectively in our current histopathology practice. 

## 2. Materials and Methods

### 2.1. Patients

A retrospective study of our surgical series of HCA was performed with the help of the Bordeaux group on thirty-one cases retrieved from the archives of our department of pathology. Six additional HCA surgically removed in our institution were then similarly studied, leading to a total number of 37 cases from January 1992 to January 2012.

Clinical data including age, sex, body mass index (BMI), and oral contraceptive (OC) use, number of lesions, and clinical presentation which led to HCA diagnosis were retrieved from the clinical files.

### 2.2. Histopathological and Immunohistochemical Analyses

Formalin-fixed paraffin-embedded liver specimens were routinely stained with Hematoxylin Eosine (HE) to identify the histopathological characteristics of each one of the HCA. Liver adenomatosis was defined when more than 10 HCA were present within the liver [13, 15, 16]. Then, immunohistochemical staining for cytokeratin 7 (CK7, monoclonal mouse antibody, 1 : 250 dilution, Dako), L-FABP (polyclonal rabbit antibody, 1 : 50 dilution, Abcam), CRP (monoclonal rabbit antibody, 1 : 1500 dilution, Abcam), SAA (monoclonal mouse antibody, 1/30 dilution, Dako), GS (monoclonal mouse antibody, 1/800 dilution, BD Bioscience), and *β*-catenin (monoclonal mouse antibody, 1/300 dilution, BD Bioscience) were performed according to the recommendations of the Bordeaux group. In cases suspicious of transformation into HCC, immunohistochemical staining of glypican-3 (monoclonal mouse antibody, 1/100 dilution, BioMosaics) was also carried out. HE and IHC staining of 31 cases was analyzed as a training set, with the help of the Bordeaux group. Based on this training, one pathologist (CS) examined subsequently the next 6 cases.

## 3. Results

The clinical data of the 37 patients are given in Table 1.

The majority of the patients in our series were women (89%). The mean age at diagnosis was 40.9 years with a range from 17 to 68 years of age. In addition, there was one case of HCA surgically removed at 11 years of age in a boy with congenital absence of the portal vein. Twenty of the 33 female patients (66.7%) were on OC. Fifteen patients (44.1%) had a BMI higher than or equal to 25 and none of the patients had diabetes. Fifteen patients (42.9%) presented with symptoms related to intrahepatic hemorrhage of HCA. The other patients were diagnosed either incidentally or during the workup for other nonrelated symptoms. The young boy was followed since several years for portal vein agenesis. HCA was single in 22 patients (59.5%), whereas 3 patients (8.1%) had liver adenomatosis.

The histological and immunohistochemical analyses allowed us to classify the HCA of our series of 37 patients into the four recently described categories (Table 2).

Lack of L-FABP expression, suggestive of *HNF1*α**-inactivated HCA (H-HCA), was observed in 9 patients (25%). All patients in this group were women, aged from 27 to 68 years; 55.5% had been on OC and 28% had a high BMI. The first manifestation was bleeding in 44.4%. Four women had a single lesion and 1 had liver adenomatosis. The size of the lesions varied from 30 to 100 mm. Followup, available for 7 patients, ranged from 10 to 129 months with all patients alive, one harboring residual HCA. 

**Figure 1:**
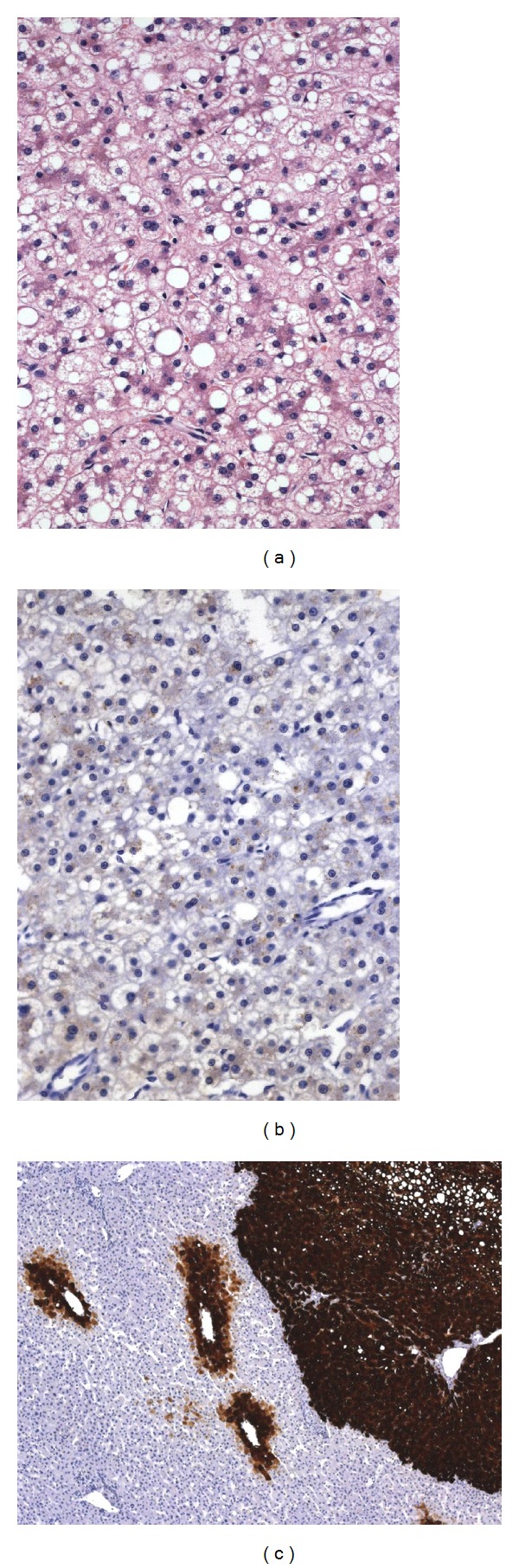
*β*-catenin-activated/*HNF1*α**-inactivated HCA ((a), Obj ×20) with negative L-FABP ((b), Obj ×20), and strong positive GS ((c), Obj ×5) staining. Note the normal GS staining around the perivenular areas of the adjacent normal liver on the left.

On HE staining, H-HCA showed a very homogeneous histological aspect. The lesion had a nodular and clear appearance at low magnification. The liver cell plates were regular and composed of large hepatocytes showing moderate-to-marked steatosis and a small dark nucleus. A few compressed liver cell plates with hepatocytes exhibiting an acidophilic cytoplasm were observed in between. There were no atypia, no inflammation, and no sinusoidal congestion. Lipofuscin pigment was abundant in some cases. The adjacent liver was normal in 8 patients and showed moderate steatosis in 1 patient. The absence of L-FABP staining was not always easy to assess. Indeed, in 2 patients, we observed more a lower L-FABP expression by contrast to the adjacent normal liver than a true negative staining. We found it therefore useful to have this internal positive control for comparison. No CRP or SAA staining was observed. Rare scattered hepatocytes expressing CK7 were present mainly around vessels or fibrotic areas. GS staining was negative or sometimes focally expressed at the borders of the adenoma or around vessels, whereas outside the lesion, GS was characteristically expressed in the perivenular areas allowing the recognition of the normal parenchyma. Importantly, one patient had a classical H-HCA showing a strong diffuse GS staining with focal nuclear staining for *β*-catenin suggesting a subgroup of *β*-catenin-activated/*HNF1*α**-inactivated HCA and was classified within the group of b-HCA (Figure 1). No HCC transformation was observed in this HCA. In one patient, the LFABP-negative HCA contained several granulomas, whereas the adjacent liver was completely normal. The patient was found to suffer from sarcoidosis. One patient had an additional lesion of focal nodular hyperplasia and one patient had multiple HCA in a context of absence of the left portal vein. The nontumoral liver in this patient was characterized by hypoplastic portal veins within the portal tracts. The five patients with multiple lesions showed the same histological and immunohistochemical features in all adenomas.

Inflammatory HCA (IHCA) with expression of both CRP and SAA were found in 20 patients (55.5%). They were 18 women and 2 men, aged from 17 to 49 years, and 50% of the women had been on OC. The BMI was high in 45% of the patients and the first manifestation was bleeding in 35%. Twelve patients had a single lesion and 2 had liver adenomatosis. The size of the lesions varied from 27 to 140 mm. Followup, available in 18 patients, ranged from 10 to 200 months. All patients are alive, two with residual HCA. 

**Figure 2:**
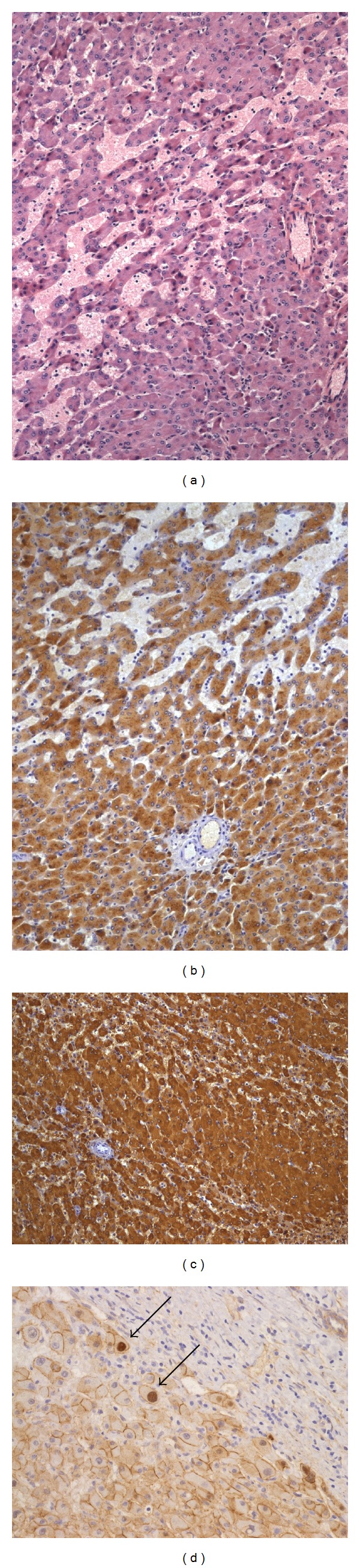
*β*-catenin-activated/inflammatory HCA ((a), Obj ×5) with strong SAA ((b), Obj ×5), and GS ((c), Obj ×5) staining showing focal nuclear *β*-catenin staining indicated by arrows ((d), Obj ×20).

In our hands, CRP was more powerful than SAA to stain IHCA. Most of the time, it was strongly and diffusely positive within the adenomas, whereas SAA showed a weaker and more granular staining. However, in some cases it was the reverse and the combination of the two staining was therefore very useful. Histologically, IHCA exhibited variable degree of macrovesicular steatosis and inflammation, whereas sinusoidal dilatation and congestion were common features. At low magnification, they were readily identified by the sinusoidal dilatation, a constant finding, and, when present, pseudoportal tracts containing inflammatory cells and delineated by a prominent ductular reaction underlined by the CK7 staining were also very characteristic. Thick-walled arteries were scattered within the lesions and, at higher magnification, nuclear atypia were often seen. Sixteen cases were very characteristic by histology alone, whereas the diagnosis was only firmly made by IHC in 4 cases. L-FABP staining was always normal and similar to the adjacent nontumoral liver. GS staining was negative or very focally expressed, adjacent to vessels or to the borders of the adenomas, by contrast to its normal perivenular expression outside the lesions. However, importantly, two cases with histological and immunohistochemical features of IHCA showed also a strong and diffuse GS expression. One of them had also a focal nuclear expression of *β*-catenin suggesting a subgroup of *β*-catenin-activated/inflammatory HCA (b-IHCA) and was classified within the group of b-HCA (Figure 2). In the second one, we were not able to identify nuclear *β*-catenin staining and we did not classify it as a b-IHCA. Two IHCA cases were associated with a lesion of focal nodular hyperplasia. In the patients harboring multiple lesions, all lesions were IHCA. The adjacent liver was completely normal in 16 patients, moderate steatosis was found in 3 patients, and no adjacent liver was available for 1 patient.

Interestingly, one 47-year-old woman (not included in Table 2) on OC and with a normal BMI was found to have two H-HCA and two IHCA by IHC. Histologically, the four lesions looked very similar and were more suggestive of IHCA, even if two of them lacked L-FABP expression. GS staining was strong in one of the H-HCA and also in one of the IHCA and we found focal *β*-catenin nuclear staining in both lesions. She is alive without recurrence at 114 months of followup.

Five patients (14%) were identified as having a *β*-catenin positive HCA (b-HCA). In this group, there were two women on OC and one without, one man who did not take any medication, and one 11-year-old boy. Four patients had a normal BMI and all had a single large lesion (50–180 mm) discovered mostly incidentally in the adults and followed for a few years in the boy because of congenital absence of the portal vein. Imaging techniques revealed absence of the left portal vein in the man. The 5 patients are all alive without recurrence or residual lesion (followup range: 8–120 months).

**Figure 3:**
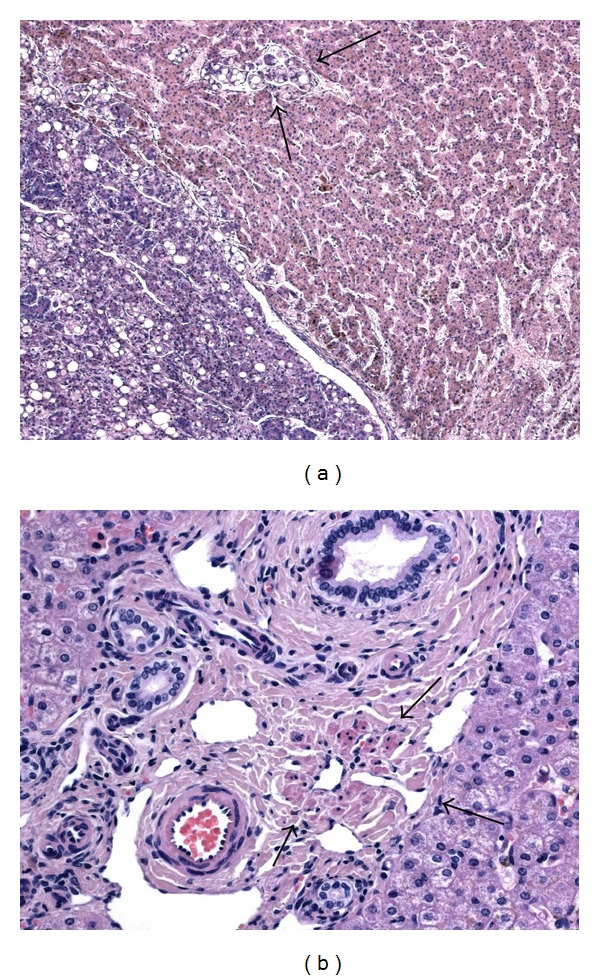
*β*-catenin-activated HCA with malignant transformation in a young boy with congenital absence of the portal vein. Microscopic view of the adenoma on the right and of the hepatocellular carcinoma on the left with vascular invasion indicated by arrows ((a), Obj ×10). The adjacent nontumoral liver shows hypoplastic portal vein branches in middle-sized portal tracts (arrows) ((b), Obj ×20).

Histologically, these HCA were variably atypical with hepatocytes of varying sizes exhibiting enlarged and irregular nuclei and with thicker liver cell plates. No significant steatosis was found. In the two male patients, GS staining was strong and diffuse and *β*-catenin nuclear staining was easy to find. The center of the b-HCA in these two patients showed transformation in HCC with rosette formation, poor differentiation, vascular invasion, and glypican-3 positivity (Figure 3). In addition, in the man, the b-HCA contained important amounts of dark and coarse Dubin-Johnson-like brown pigment that was stained both by PAS and Fontana-Masson. No adjacent liver was available in the man, whereas in the child it showed remodeling of the liver architecture together with absent or hypoplastic portal vein branches within the portal tracts (Figure 3). In one woman, the HCA showed heterogeneous patchy GS staining and the nuclear positivity of *β*-catenin was difficult to find requiring the use of several slides. Many vessels were present within the lesion, together with very thick liver cell plates and obvious nuclear irregularities. Staining for CK7 was positive in numerous cells and glypican-3 was negative. In these 3 case of b-HCA, there was no expression of inflammatory proteins, except in a few scattered cells and L-FABP staining was normal. As already mentioned, one case of b-HCA showed the histological and immunohistochemical characteristics of an H-HCA (Figure 1) and another b-HCA, those of an IHCA (Figure 2). There were less cytological atypia in these two cases and it is only the diffuse and strong GS staining (Figures 1(c) and 2(c)) that warned us to search for *β*-catenin nuclear positivity which was very focal (Figure 2(d)).

The last two patients in our series remained unclassified. Their HCA showed a normal L-FABP staining and no staining for SAA, CRP, *β*-catenin, and GS. They were both found in females, with a mean age of 30 years at presentation. Both were on oral contraceptives and had a high BMI. They presented with hemorrhage complicating their HCA. One of them had a single lesion and one of them had multiple lesions, all the same histologically. Both are alive without recurrence or residual lesion at 24 and 83 months, respectively. 

**Figure 4:**
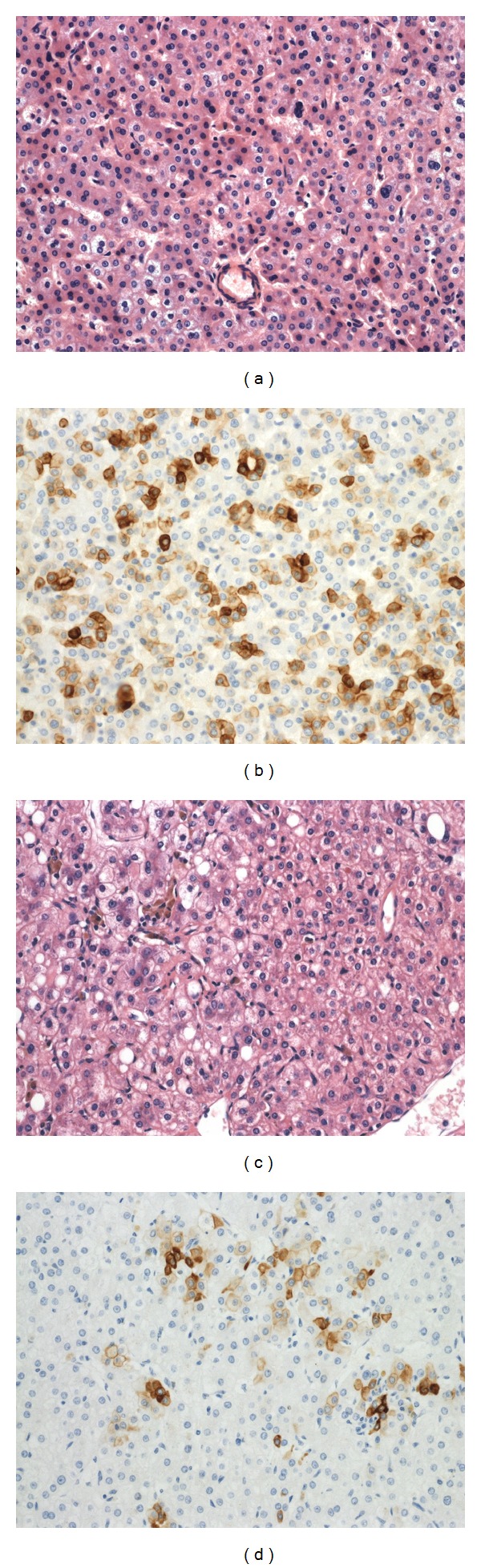
Unclassified HCA (a) with numerous small hepatocytes expressing CK7 (b). Unclassified HCA with worrisome nuclear atypia (c) with numerous small hepatocytes expressing CK7 (d). (Obj ×20).

One HCA was composed of small hepatocytes arranged very regularly in thin or slightly enlarged liver cell plates and frequently expressing CK7, whereas the other one showed a highly atypical HCA with large liver cell plates, numerous thick-walled vessels, some steatosis, and worrisome nuclear atypia, with numerous small hepatocytes in between expressing CK7 (Figure 4). This last case was very similar to the one of the woman with b-HCA, except for the absence of GS and *β*-catenin nuclear staining. The adjacent liver showed mild steatosis in the first patient and was normal in the second one.

## 4. Discussion

Light has been recently shed on the genotype/phenotype correlation in hepatocellular adenoma (HCA) leading to the identification of four different subtypes: *HNF1*α**-inactivated HCA (H-HCA), inflammatory HCA (IHCA), *β*-catenin activated HCA (b-HCA), and unclassified HCA [9–15]. This new classification has been validated [17, 18] and is now included in the 2010 edition of the WHO classification of Tumours of the Digestive System [19]. By reviewing our surgical series of HCA, we learned how to recognize histopathologically the different subtypes and how to perform and interpret adequately the immunohistochemical staining of these lesions. This learning experience allowed us to classify our retrospective series of 37 patients who underwent HCA surgical resection in our institution.

The clinical characteristics and the frequency of each subtype in our series are similar to what is described in the Western literature [13, 17, 18]. Indeed, we confirmed not only that HCA still occur more frequently in women on oral contraception but also that it can be found in men and in this case be either IHCA or b-HCA. Bleeding is still a frequent complication of HCA that leads to the diagnosis but our study shows that HCA are more and more often found incidentally, probably thanks to the progresses of imaging techniques.

In our series, as in others [13, 17, 18], the larger group of HCA corresponded to the IHCA and this group was more frequently associated with a high BMI. Also in concordance with published data, we saw that multiple lesions were more frequent in the H-HCA subgroup and that b-HCA were large, single adenomas that carry a higher risk of malignant transformation [20], as demonstrated by the presence of HCC in two of our cases. We also confirmed that *β*-catenin nuclear accumulation can be found in otherwise typical IHCA leading to a subgroup of b-IHCA [15]. Interestingly, we made the same observation in H-HCA, a phenomenon not well known in the literature so far. To understand better this particular finding, molecular studies are required in these cases. 

In our experience, most of the HCA were easily classified into one of the subtypes using immunohistochemistry. However, interpretation of the staining requires comparison with nontumoral liver tissue and we would like to strongly recommend having this internal control within the same block. Indeed, L-FABP negative staining in H-HCA can be difficult to assess in the absence of adjacent normal liver tissue. By contrast, CRP can be present in scattered normal hepatocytes outside the lesion. Regarding the identification of IHCA, CRP detection was commonly easier to interpret than SAA because of being strong and diffuse, but for some of the cases it was the reverse and so both stainings are probably useful to identify IHCA when starting to study these lesions. HCA borders are not always easy to identify and GS staining is very helpful to allow the recognition of the normal tissue by labeling the perivenular areas. GS staining is also mandatory to recognize b-HCA in which it is commonly strongly and diffusely expressed. This is extremely helpful knowing that *β*-catenin nuclear staining can instead be very focal and difficult to find. As exemplified by one of our b-HCA, GS staining can also be patchy within the lesion and this has also to be considered as an abnormal staining suspicious for b-HCA, as shown previously by the Bordeaux group [15, 21]. However, although this type of GS staining is worrisome, its molecular background is still under investigation. Importantly, a focal GS staining limited to the HCA borders or to perivenular areas within the lesion is not considered as abnormal. In this series, four women showed b-HCA, either isolated or combined with IHCA or H-HCA. Three had a strong diffuse GS staining and one had the patchy GS staining already mentioned. In all but one of these patients, the nuclear labeling for *β*-catenin was very focal and required the staining of several slides before identification. Therefore, at least in case of a strong diffuse GS staining, *β*-catenin IHC has to be performed in several slides to identify a b-HCA. Regarding the patchy GS staining, no clear recommendation can be made so far.

Knowing all these possible pitfalls of immunohistochemistry will help the pathologist confronted to the biopsies that will probably be more and more often done in HCA in the next few years before surgical resection. Indeed, identification of the different types of HCA led to new propositions of management of these lesions, including performing a liver biopsy either because the nature of the lesion is uncertain on imaging techniques or to subtype an HCA prior to decide on the appropriate treatment [15, 17, 21–24]. A recent study by the Beaujon group demonstrated the accuracy of classifying HCA by a combination of magnetic resonance imaging and liver biopsy [22] and decision-making models involving liver biopsies are proposed by several authors [21, 23]. To interpret adequately biopsy specimen, it is mandatory to obtain both tumoral and nontumoral tissue for the comparison of IHC staining and to keep in mind that GS staining can be more helpful in identifying b-HCA than nuclear *β*-catenin positivity that can be very focal [21]. 

In our series there were some interesting observations of associations between HCA and a particular clinical context. The most interesting one was the curious coincidence observed in 3 patients. A male child and two adults, one female and one male, were found to have an absence of, respectively, the portal vein in the child and the left branch of the portal vein in the two adults. These findings were also associated with a hyperarterialization of the corresponding branch of the hepatic artery. Both males had a single b-HCA transformed in HCC and the female had multiple H-HCA. Focal liver cell lesions including HCA, HCC, focal nodular hyperplasia, and nodular regenerative hyperplasia have been reported in association with congenital absence of the portal vein, also known as congenital extrahepatic portosystemic shunt or Abernethy malformation [5–8, 25–27]. It has been suggested that the decreased venous blood flow combined with an increased arterial blood flow could result in abnormal perfusion of the liver and to uneven vascular perfusion of the liver giving rise to a hyperplastic response, and to a spectrum of nodular lesion including HCA [5, 28]. A study on the complications of congenital portosystemic shunts in children has shown the presence of 13 tumors in 17 patients (76%), 4 corresponding to HCA, one transformed in HCC [26]. Interestingly, by closure of the shunt and restoration of the blood flow, the authors observed a complete or partial regression of the tumors, including regression of one of the HCA. Another recent work examined specifically the liver histopathological lesions that can be found in case of congenital extrahepatic portosystemic shunts in 5 patients [27]. They found two patients with HCC and they described that the nontumoral liver was characterized by absence of portal vein in small portal tracts, absence or hypoplasia of portal vein in medium-sized portal tracts, and remodeling of the liver architecture, all features that we also found in our young patient. In the two adult cases, it is difficult to know whether anomalies of the portal venous system predisposed to HCA or if HCA resulted in some thrombosis and atrophy of the left portal venous system. In the man, no liver parenchyma outside the lesion was available, but in the woman we observed similar anomalies in the portal tracts of the normal parenchyma than those described and observed in case of congenital absence of the portal vein. The concept of anomalous portal tract syndrome [28] has been proposed as a single unifying etiological factor underlying the development of the different types of liver nodules in the context of vascular abnormalities, although for HCA the mechanisms are still poorly understood.

In this series, we also found that one patient had granulomas within her H-HCA leading to a diagnosis of sarcoidosis later on. The presence of granulomas has been described in adenomas [21], but to our knowledge the association with sarcoidosis has not been reported yet. Finally, one patient with b-HCA transformed into HCC showed a pigmented lesion, a very rare phenomenon recently reviewed by the Mount Sinai group who described a middle-aged man with a pigmented b-HCA transformed into HCC with, as in our case, an absence of the left portal vein [29]. This pigment is considered as Dubin-Johnson-like and is stained black with Fontana-Masson whereas iron staining remains negative [30]. 

In conclusion, using the new histological and immunohistochemical criteria enabled us to classify accurately our retrospective surgical series of HCA with recognition of *β*-catenin activated HCA. Our series confirms the higher risk of malignant transformation of this particular subtype and underlines the association between HCA and vascular abnormalities. Based on this learning experience that is reproducible in other series of surgically treated HCA, our recommendations to other pathologists would be (1) to always study in parallel the tumoral and nontumoral liver tissue and (2) to perform GS staining on all cases and rely on strong and diffuse GS staining to identify the potential b-HCA that requires further immunohistochemical and/or molecular confirmation. So far, a patchy GS staining is worrisome, but because it is still poorly understood, it requires further molecular investigations before giving any advice.

## Figures and Tables

**Table 1:** Clinical data.

Age (years)	11–68
Sex	
Female	33 (89%)
Male	4 (11%)
BMI (kg/m^2^)	
<25	19 (55.9 %)
≥25	15 (44.1%)
Unknown	3
OC (33 women)	
OC use	20 (66.7%)
No OC use	10 (33.3%)
Unknown	3
Number of HCA (radiological)	
Single	22 (59.5%)
Multiples	12 (32.4%)
Adenomatosis	3 (8.1%)
Symptoms	
Bleeding	15 (42.9%)
Aspecific/incidental finding	20 (57.1%)
Unknown	2

**Table 2:** Clinical data according to hepatocellular adenoma subtypes. (The patient with both H-HCA and IHCA is not included in the table.)

Characteristics	H-HCA	IHCA	b-HCA	Unclassified
Number of cases	9 (25 %)	20 (55.5%)	5 (14%)	2 (5.5%)
Age (years)	27–68	17–49	11–42	28, 32
BMI (kg/m^2^)				
<25	6	8	4	0
≥25	3 (28%)	9 (45%)	1 (20%)	2 (100%)
Unknown	0	3	0	0
Sex				
Females	9	18	3	2
Males	0	2	2	0
Number of HCA (radiological)				
Single	4 (44.4%)	12 (60%)	5 (100%)	1 (50%)
Multiple	5 (1 adenomatosis)	8 (2 adenomatosis)	0	1
Symptoms				
Bleeding	4 (44.4%)	7 (35%)	1 (20%)	2 (100%)
Aspecific/incidental finding	5	11	4	0
Unknown		2		
OC use (32 women)				
Yes	5 (55.5%)	10 (50%)	2 (67%)	2 (100%)
No	3	6	1	
Unknown	1	2		
Size of lesions (mm)	30–100	27–140	50–180	66–100
Followup (range in months)	10–129	10–200	8–120	24–83
Alive without recurrence	6	16	5	2
Alive with recurrence				
Alive with residual HCA	1	2		
Unknown	2	2		
